# The SFB/TRR 393 Collaborative Research Centre: trajectories of affective disorders: Cognitive–emotional mechanisms of symptom change

**DOI:** 10.1007/s00115-025-01886-8

**Published:** 2025-09-12

**Authors:** Tilo Kircher, Nina Alexander, Michael Bauer, Udo Dannlowski, Ulrich W. Ebner-Priemer, Philipp Kanske, Markus Wöhr, Andrea Pfennig, SFB/TRR 393 consortium, Judith Alferink, Nadine Bernhardt, Carsten Culmsee, Stefan Ehrlich, Irina Falkenberg, Katharina Förster, Andreas Forstner, Joachim Groß, Tim Hahn, Stefan Hofmann, Hamidreza Jamalabadi, Andreas Jansen, Kay Jüngling, Markus Junghöfer, Luisa Klotz, Elisabeth Leehr, Julia Martini, Susanne Meinert, Eva Mennigen, Ralph Müller-Pfefferkorn, Igor Nenadić, Carmine Pariante, Winfried Rief, Philipp Ritter, Michael Smolka, Frederike Stein, Benjamin Straube, Ida Wessing, Allan Young, Michael Ziller

**Affiliations:** 1 Universitätsklinik für Psychiatrie und Psychotherapie, Fachbereich Medizin, UKGM, Universität Marburg, Rudolf-Bultmann-Straße 8, 35039 Marburg, Germany; 2 Universitätsklinikum Carl Gustav Carus, Klinik und Poliklinik für Psychiatrie und Psychotherapie, Medizinische Fakultät, Technische Universität Dresden, Dresden, Germany; 3 Medizinische Fakultät, Institut für Translationale Psychiatrie, Universität Münster, Münster, Germany; 4 Bielefeld University, Medical School and University Medical Center OWL, Protestant Hospital of the Bethel Foundation, Department of Psychiatry, Bielefeld, Germany; 5 Fakultät für Geistes- und Sozialwissenschaften, Institut für Sport und Sportwissenschaft, Karlsruher Institut für Technologie, Karlsruhe, Germany; 6 Department of Psychiatry and Psychotherapy, Central Institute of Mental Health, University of Heidelberg, Medical Faculty Mannheim, Mannheim, Germany; 7 Institut für Klinische Psychologie und Psychotherapie, Technische Universität Dresden, Dresden, Germany; 8 Fachbereich Psychologie, Arbeitsbereich Allgemeine und Biologische Psychologie, Universität Marburg, Marburg, Germany; 9 Faculty of Psychology and Educational Sciences, Research Unit Brain and Cognition, KU Leuven, Leuven, Belgium; 10 Leuven Brain Institute, KU Leuven, Leuven, Belgium

**Keywords:** Major depressive disorder, Bipolar disorder, Course of illness, Symptom changes, MRI, Major Depression, Bipolare Störung, Krankheitsverlauf, Symptomveränderungen, MRI

## Abstract

Major depressive disorder (MDD) and bipolar disorder (BD) are prevalent and disabling psychiatric disorders, often following a chronic and relapsing course. The Collaborative Research Centre 393 (SFB/TRR 393), funded by the German Research Foundation (DFG), aims to identify trajectories and symptom changes in MDD and BD, with a focus on cognitive–emotional mechanisms and their neurobiological underpinnings.

Our research initiative seeks to (1) identify individual trajectories of recurrences and remissions in affective disorder (AD), (2) determine cognitive–emotional mechanisms and neurobiological correlates of acute symptom changes, and (3) probe mechanism-based interventions.

These goals will be pursued through a threefold approach: (1) *Continuous mobile assessment in a prospective cohort*: We will combine in-depth clinical characterization with multilevel neuroimaging, biobanking, and -omics analyses in 1500 AD patients and healthy participants over a 2-year follow-up (German Mental Health Cohort, GEMCO) at three time points. Participants will be drawn from existing DFG FOR 2107 and BMBF Early-BipoLife cohorts (Domain A). (2) *Identification of key cognitive-emotional mechanisms*: We will study emotion regulation, expectation, social cognition, and cognitive–behavioural rhythms, and their neurobiological correlates mediating symptom changes, using parallel human studies and animal experiments (Domain B). (3) *Targeted interventions*: We will probe key cognitive–emotional mechanisms in relation to recurrences and remissions (Domain C).

Over a 12-year period, we will elucidate environmental, psychosocial, and (neuro)biological predictors of illness course; cognitive–emotional and neurobehavioural mechanisms underlying real-life recurrences and remissions; and targeted, mechanism-based interventions.

## Abstract (de)

Depressive (MDD) und bipolare Störungen (BD) sind weit verbreitete und schwerwiegende psychische Störungen, die häufig chronisch oder rezidivierend verlaufen. Der von der Deutschen Forschungsgemeinschaft (DFG) geförderte Sonderforschungsbereich 393 (SFB/TRR 393) zielt darauf ab, Krankheitsverläufe und Symptomveränderungen bei affektiven Störungen (AD) zu identifizieren, wobei der Schwerpunkt auf kognitiv-emotionalen Mechanismen und deren neurobiologischen Grundlagen liegt. Unsere Forschungsinitiative zielt darauf ab: 1. individuelle Verläufe von Rezidiven und Remissionen bei AD zu identifizieren; 2. kognitiv-emotionale Mechanismen und deren neurobiologische Korrelate zu bestimmen, und 3. mechanismusbasierte Interventionen zu erproben. Diese Ziele werden durch einen 3‑fachen Ansatz verfolgt: *1. Kontinuierliche mobile Erfassung in einer prospektiven Kohorte*: Wir werden eine eingehende klinische Charakterisierung mit Neuroimaging‑, Biobanking- und -omics-Analysen bei *n* = 1500 AD-Patienten und gesunden Probanden über einen 2‑jährigen Follow-up-Zeitraum (German Mental Health Cohort, GEMCO) zu 3 Zeitpunkten durchführen. Die Teilnehmer werden aus den bestehenden DFG-FOR-2107- und BMBF-Early-BipoLife-Kohorten (Domäne A) rekrutiert. *2. Identifizierung kognitiv-emotionaler Mechanismen*: Wir werden Emotionsregulation, Erwartungshaltung, soziale Kognition und kognitiv-verhaltensbezogene Rhythmen sowie deren neurobiologische Korrelate anhand paralleler Humanstudien und Tierversuche untersuchen (Domäne B). *3. Etablierung gezielter Interventionen*: Wir werden kognitiv–emotionale Mechanismen in Bezug auf Rezidive und Remissionen erproben (Domäne C). Ziel ist es, über einen Zeitraum von 12 Jahren umweltbedingte, psychosoziale und (neuro)biologische Prädiktoren des Krankheitsverlaufs, kognitiv-emotionale and neurobehaviorale Mechanismen, die Rezidiven und Remissionen im realen Leben zugrunde liegen, sowie gezielte, mechanismusbasierte Interventionen aufzuklären.

Major depressive disorder (MDD) and bipolar disorder (BD) are highly prevalent psychiatric illnesses marked by episodic and often chronic courses. They significantly impact individuals and society, with lifetime prevalence rates estimated between 6% and 12% in Europe [14]. These disorders typically manifest during early adulthood and follow heterogeneous courses.

Approximately 70–90% of patients experience multiple episodes [1]. While some patients may experience remission, others develop chronic courses with substantial psychosocial and cognitive impairments [8, 9]. Functional deficits extend beyond acute episodes, highlighting the need for a trajectory-based approach to understanding and treating affective disorders (ADs; [16]).

Despite advances in understanding acute episodes and treatment responses, long-term disease trajectories remain poorly understood. Symptoms often reoccur or persist subsyndromally, contributing to functional impairment, reduced quality of life, and increased societal burden. The SFB/TRR 393 consortium addresses this gap by investigating the dynamic and individualized trajectories of ADs, integrating real-time assessments via mobile phones, mechanistic studies, and targeted interventions ([4, 5, 10, 12]; Figs. 1, 2, 3 and 4).

**Fig. 1:**
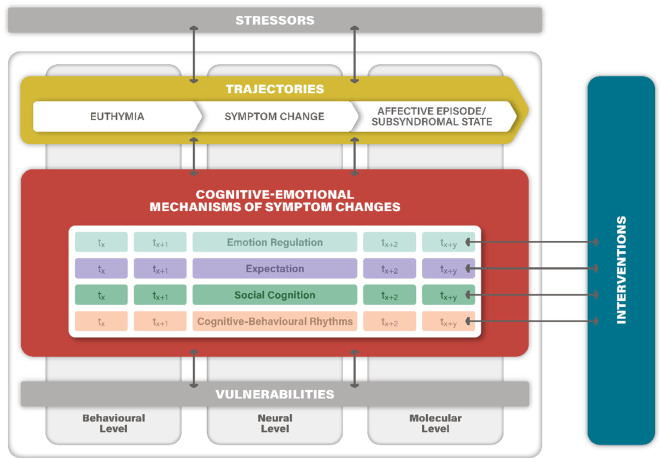
Model of disease trajectories in ADs and levels of their investigation in the SFB/TRR 393. The four key cognitive–emotional mechanisms of emotion regulation, expectation, social cognition, and cognitive–behavioural rhythms are implicated in symptom changes and trajectories of affective disorder; t_x_, t_x+1_, etc. represent changes in the mechanisms across time, with multiple time points of assessment in the GEMCO and the SFB/TRR 393 projects. Interventions are targeted to address particular cognitive–emotional mechanisms. Biopsychosocial vulnerabilities and stressors modulate these mechanisms and trajectories. In rodent models, specific cognitive–emotional mechanisms, vulnerabilities, and stressors can be manipulated. Applying a holistic perspective, in longitudinal human and animal studies changes must be analysed on multiple levels (behavioural, neural, molecular). The three Domains of the planned SFB/TRR 393 are colour coded: Trajectories (Domain A), Mechanisms (Domain B), and Interventions (Domain C). (© T. Kircher)

## Key objectives of SFB/TRR 393

The SFB/TRR 393 research programme is guided by the following primary objectives:

- To identify stressors and modifying factors impacting the trajectories and symptom changes of MDD and BD in real life by characterizing their clinical, behavioural, neurobiological, and molecular basis (Research Domain A).
- To uncover cognitive–emotional and neurobiological mechanisms of symptom changes, including transitions between stability, recurrence, and remission (Research Domain B).
- To modify specific cognitive–emotional pathomechanisms aimed at personalized and preventive interventions (Research Domain C).

These objectives are pursued through a highly interdisciplinary and translational design, incorporating both longitudinal and experimental human studies and animal models as well as capitalizing on cutting-edge technology and analytics [4, 5, 10, 12].

## The German Mental Health Cohort—GEMCO

**Fig. 2:**
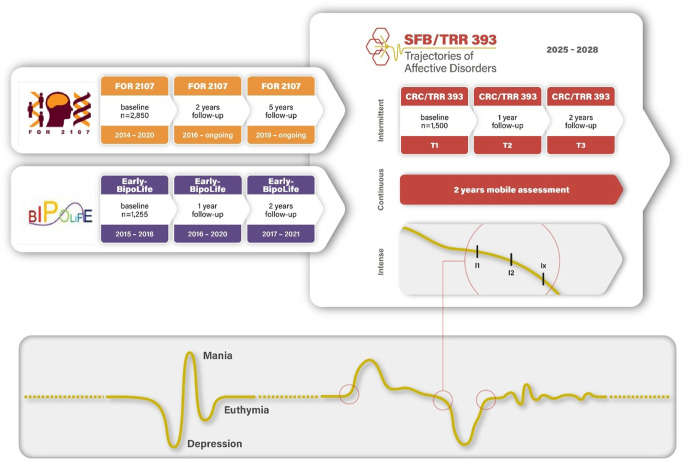
Recruitment of German Mental Health Cohort (GEMCO) participants for Domains A, B, and C through existing cohorts (DFG FOR 2107, BMBF Early-BipoLife) and data sampling: intermittent sampling at baseline, and 1‑year and 2‑year follow-up; continuous sampling via mobile assessment; and intense sampling during times of symptom changes/inflection signals. (© T. Kircher)

To support these objectives, SFB/TRR 393 has established the German Mental Health Cohort (GEMCO), a deeply phenotyped longitudinal sample comprising 1500 participants (Fig. 2). GEMCO integrates participants from two previous national initiatives—DFG FOR 2107 (*n* = 3200 MDD, BD, schizoaffective disorder [SZA], schizophrenia [SZ], and healthy control participants with deep phenotyping and 2‑ and 5‑year longitudinal follow-up; [11]) and BMBF Early-BipoLife (*n* = 1200 at-risk youth cohort with neuroimaging and 2‑year follow-up; [15]); thus, with already available baseline assessments of more than 4000 participants, including clinical, magnetic resonance imaging (MRI), genome-wide association study (GWAS), cognitive, and other deeply phenotyped measures. This offers a comprehensive resource for the current GEMCO, which follows up these participants after 10, 11, and 12 years (GEMCO sample: MDD *n* = 900, BD *n* = 300, and healthy controls *n* = 300). The GEMCO is assessed using both intermittent deep phenotyping (baseline, 1‑year, and 2‑year follow-up), continuous digital monitoring, and intensive phenotyping allowing for unprecedented insight into the temporal dynamics of AD symptoms and their underlying cognitive–emotional and neurobiological mechanisms (Fig. 2; [4]).

## Disease trajectories and symptom change

A core concept in SFB/TRR 393 is the identification of “inflection signals”—symptom changes that precede full-blown episodes or mark the onset of remission (Fig. 2). These signals are captured using continuous digital phenotyping via smartphone-based assessments [4, 5]. This approach makes it possible to detect early warning signs in real time, enabling the invitation of patients into the laboratory during inflection signals/symptom changes for mechanistic investigations (Domain A and B projects [5, 12]) and timely, personalized interventions (Domain C projects) [10].

### Infobox 1 Definition of central SFB/TRR 393 concepts

**Symptom change:** Change of AD symptoms during a specific time period. This change may precede subsyndromal fluctuation or may develop into an episode or indicate imminent remission (see Fig. 2). Relevant “symptom changes” are operationalized as “inflection signals”.

**Inflection signal:** An inflection signal is a (clinically) relevant symptom change over time detected by mobile assessment in humans (Fig. 2) or continuous, longitudinal behavioural observation in animals.

**Course of illness:** Psychopathological, psychosocial, and functional course over years in an individual patient.

**Disease trajectories:** Typical, data-driven defined courses of illness over years in particular groups of patients.

## Structure of the SFB/TRR 393

**Fig. 3:**
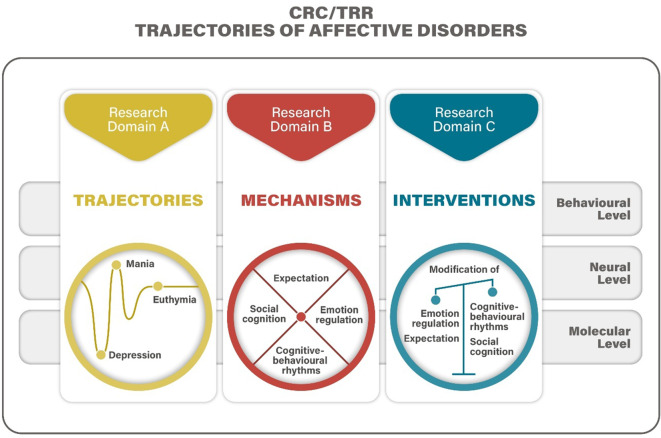
General framework of SFB/TRR 393. (© T. Kircher)

**Fig. 4:**
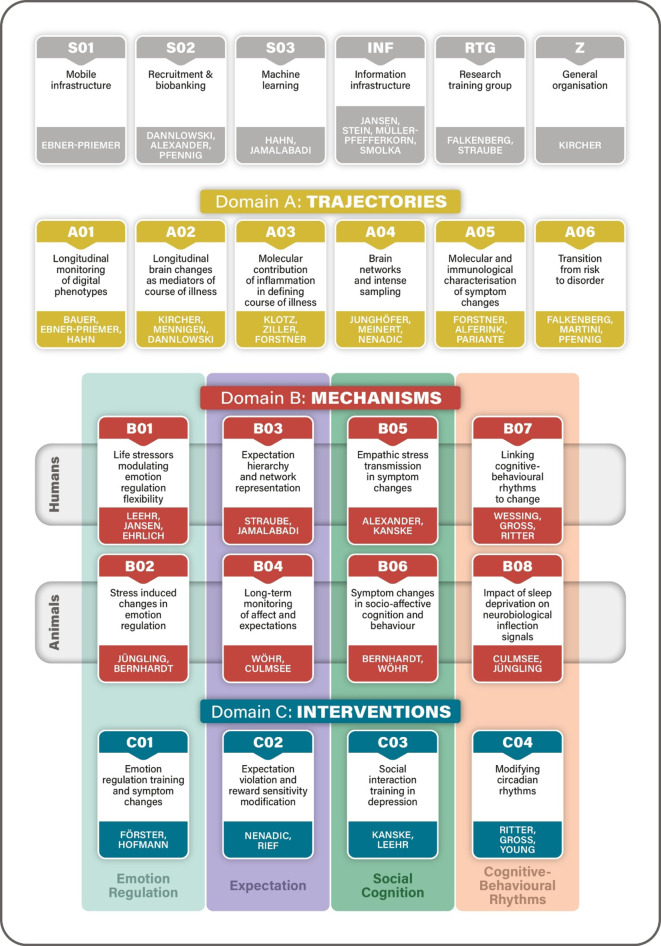
Overview of SFB/TRR 393 projects. The Research Domains A—Trajectories, B—Mechanisms and C—Interventions, the cognitive–emotional mechanisms (Emotion Regulation, Expectation, Social Cognition, and Cognitive–Behavioural Rhythms) and the central infrastructure projects (grey boxes). Each of the four cognitive–emotional mechanisms links a human Domain B and animal Domain B translational project with one Domain C Intervention project. The four cognitive–emotional mechanisms are also probed in the Domain A Trajectories projects and in the GEMCO (German Mental Health Cohort, implemented in S01, S02, INF together with all other human projects). (© T. Kircher)

The SFB/TRR 393 is organized into three interwoven research domains—Trajectories (A), Mechanisms (B), and Interventions (C)—and is supported by central infrastructure projects (Figs. 3 and 4).

- *Domain A: Trajectories* focuses on real-life longitudinal monitoring and symptom detection. Projects include continuous digital phenotyping, structural and functional neuroimaging, immune and molecular marker studies, and identifying early transitions from risk to disorder [5].
- *Domain B: Mechanisms* investigates the cognitive–emotional pathways influencing symptom changes. Projects target emotion regulation, expectation, social cognition, and circadian rhythms across behavioural, neural, and molecular levels using both human experiments and translational animal models [10].
- *Domain C: Interventions* tests novel, mechanism-based therapeutic approaches tailored to the four identified mechanisms. This includes emotion regulation training, expectation modification, social stress management, and chronotherapy [12].
- *Central Projects:* S01: Mobile infrastructure; S02: Recruitment, phenotyping, and biobanking; S03: Machine learning and modelling; INF: Data integration, sharing, and Open Science; RTG: Doctoral training program; Z: Administrative coordination, public outreach [4].

The entire consortium works synergistically, with data and insights flowing between domains and infrastructure projects. The integrated structure ensures that findings in basic science (Domain B [12]) inform longitudinal observational work (Domain A [5]) and feed directly into intervention designs (Domain C [10]).

## Cognitive–emotional mechanisms of symptom change

SFB/TRR 393 focuses on four key cognitive–emotional mechanisms implicated in the onset and course of MDD and BD. SFB/TRR 393 integrates paradigms in both human and animal studies to assess these dynamics during (disease) trajectories [12].

### Emotion regulation.

**Fig. 5:**
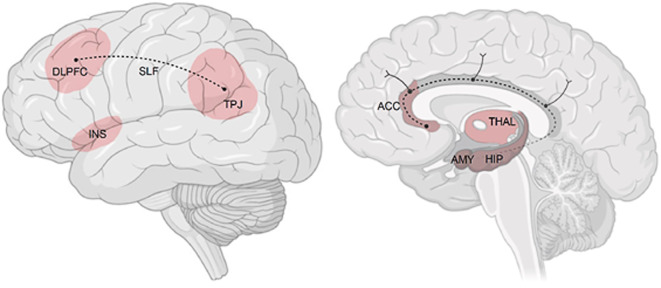
Brain networks associated with (1) affective disorder (AD) in cross-sectional structural and functional magnetic resonance imaging studies; (2) disease trajectories in AD; and (3) cognitive–emotional mechanisms (emotion regulation, expectation, social cognition, and cognitive–behavioural rhythms) hypothesized to interact with periods of symptom change. *DLPFC* dorsolateral prefrontal cortex, *INS* (anterior) insula, *TPJ* temporoparietal junction, *ACC* anterior cingulate cortex, *THAL* thalamus, *AMY* amygdala, *HIP* hippocampus, *SLF* superior longitudinal fasciculus. (© T. Kircher, F. Stein)

Emotion regulation (ER) encompasses automatic and volitional processes for modulating emotional responses. Patients with MDD and BD commonly exhibit ER deficits, including inflexible strategy use and maladaptive responses. Emerging models emphasize ER flexibility—the ability to adapt strategies contextually—as critical to mental health. Neural correlates include a fronto-limbic network involving the amygdala, anterior cingulate cortex (ACC), and dorsolateral prefrontal cortex (DLPFC; Figs. 1, 3, 4 and 5).

### Expectation.

Expectations, both explicit and implicit, influence perception and behaviour. In MDD, dysfunctional negative expectations about self and future contribute to symptom persistence. Alterations in reward learning and expectation updating are linked to affective episodes. Neural mechanisms involve the DLPFC, ACC, and limbic structures (Figs. 1, 3, 4 and 5). Experimental paradigms in SFB/TRR 393 probe expectation formation, updating, and violation during symptom change.

### Social cognition.

Social cognition, including empathy and theory of mind, is often impaired in ADs. SFB/TRR 393 investigates how empathic stress transmission—witnessing others’ stress—affects symptom dynamics. Brain regions such as the anterior insula and temporoparietal junction are implicated (Figs. 1, 3, 4 and 5). Social interaction training is evaluated as a potential intervention to enhance resilience and reduce stress-induced symptom change.

### Cognitive–behavioural rhythms.

Disruptions in circadian rhythms and sleep are among the earliest signs of affective episodes. SFB/TRR 393 examines how altered behavioural patterns, such as sleep–wake cycles and activity rhythms, predict and mediate symptom changes. Chronotherapeutic interventions, including wake therapy, are evaluated for their capacity to reset these rhythms and induce remission (Figs. 1, 3, 4 and 5).

### Infobox 2 Further information

- www.sfb-trr393.de
- www.for2107.de
- www.bipolife.org

## Neurobiological correlates

### Brain imaging.

Longitudinal neuroimaging studies are essential for understanding structural and functional changes associated with illness trajectories. Cross-sectional studies suggest volume loss and connectivity alterations in cortico-limbic networks in both MDD and BD (Fig. 5; [3]). However, few longitudinal studies exist [6, 17]. SFB/TRR 393 addresses this gap by acquiring MRI data at multiple time points, linked to real-time symptom monitoring (Fig. 2).

### Molecular neurobiology.

Genetic and epigenetic mechanisms modulate vulnerability and resilience in MDD and BD. SFB/TRR 393 integrates findings from genome-wide association studies (GWAS) and transcriptomics. Special attention is given to the top-ranked cross-disorder risk gene *CACNA1C*, implicated in calcium signalling and immune regulation [13]. Genetic *Cacna1c* mouse and rat models are used to investigate gene–environment interactions relevant to symptom dynamics.

### Immunological mechanisms.

Neuroinflammation plays a role in the pathophysiology of ADs [2]. SFB/TRR 393 explores immune signatures linked to treatment resistance and recurrence. Studies assess cytokine profiles, microglial activation, and immune–metabolic changes during symptom transitions, both in humans and animal models.

### Animal models.

Improved rodent models for human mental disorders and treatments are needed to advance brain science [7]. Our optimized behavioural phenotyping approach, combining continuous and intermittent long-term monitoring of behaviour and ultrasonic vocalizations, helps detect relevant behavioural changes tied to disease progression. This method allows us to identify and manipulate cognitive–emotional mechanisms and their neurobiological bases. By integrating high-resolution data and allowing animals to exhibit more natural behaviours in semi-natural environments, we enhance the sensitivity for detecting key alterations. This approach aligns with the 3R principles of animal research, improves animal welfare, and reduces the number of animals required.

## Methodological innovations

### Continuous digital phenotyping.

Using smartphone-based adaptive e‑diaries, SFB/TRR 393 collects real-time data on mood, behaviour, and context. These data feed into algorithms detecting inflection signals. Participants are prompted for expert interviews and intensive assessments following signal detection. This method enhances temporal resolution and ecological validity.

### Translational animal models.

Parallel rodent studies allow for experimental manipulation of stress, emotion, and social context. Long-term behavioural and electrophysiological monitoring enables detection of animal analogues to human inflection signals. Findings inform mechanistic hypotheses in human studies.

### Machine learning for prediction.

Machine learning is employed to model trajectories and predict recurrence based on multimodal data. SFB/TRR 393 addresses challenges such as data heterogeneity and interpretability by combining digital phenotyping with neurobiological markers. Machine learning tools are designed to inform personalized interventions.

### Mechanism-based therapies.

Intervention studies in SFB/TRR 393 target identified cognitive–emotional mechanisms:

- ER training to improve strategy flexibility.
- Expectation modification techniques to reduce negative biases.
- Social cognition training to mitigate stress contagion.
- Chronotherapeutics to stabilize behavioural rhythms. These approaches are assessed for their efficacy in preventing recurrence and promoting sustained remission.

### Personalization and contextualization.

By integrating real-time data and individual risk profiles, SFB/TRR 393 aims to tailor interventions dynamically. This approach acknowledges intra-individual variability and situational context as critical to treatment success.

### Multilevel integration and interdisciplinary collaboration.

The consortium brings together psychiatry, neuroscience, psychology, molecular biology, and data science. It employs a bidirectional translational model where findings from basic science inform clinical applications and vice versa. The three domains—A (Trajectories), B (Mechanisms), and C (Interventions)—are tightly integrated to enable cross-fertilization of methods and insights (Fig. 4, see [4, 5, 10, 12] for more details).

## Research training group

Interdisciplinary research in the CRC/TRR 393 is important as expertise in different disciplines is necessary for acquiring a doctoral degree (PhD/MD). The integrated RTG will provide a platform for the structured training of CRC/TRR 393 doctoral candidates. It will build on the excellent existing university structures and RTGs/IRTGs of the three partner sites and provide a sophisticated training programme specific to the CRC/TRR 393 topics. This will enable our doctoral candidates to master their thesis projects with excellence on an internationally competitive level. Key elements of the RTG will be (1) lectures covering all domains of the CRC/TRR 393; (2) workshops; and (3) training sessions covering scientific techniques and key skills regarding laboratory management; (4) specific laboratory visits for (international) networking and knowledge acquisition; and (5) a structured, continuous supervision. All this will be complemented by student retreats and journal clubs, method workshops and soft skill training camps, which will be provided in collaboration with established local early-career structures (see [4] for more details).

## Conclusion and outlook

SFB/TRR 393 represents a shift toward dynamic, mechanism-based psychiatry for MDD and BD. By combining continuous phenotyping, experimental neuroscience, and targeted interventions, it strives to unravel the complexity of AD trajectories. Its outcomes may redefine diagnostic and therapeutic standards, paving the way for precision psychiatry based on real-world, individualized data.

## Practical conclusion

- Individual courses of illness are not predictable.
- An individualized treatment plan is obligatory.
- Treatment guidelines should be used as a framework.

## Declarations

For this article no studies with human participants or animals were performed by any of the authors. All studies mentioned were in accordance with the ethical standards indicated in each case.
